# Phytomonitoring and Phytoremediation of Environmental Pollutants

**DOI:** 10.3390/plants13030366

**Published:** 2024-01-26

**Authors:** Maria Cristina Sorrentino, Simonetta Giordano, Valeria Spagnuolo

**Affiliations:** 1 Council for Agricultural Research and Economics, Cereal and Industrial Crops (CREA-CI), Via Torrino 2, 81100 Caserta, CE, Italy; mariacristina.sorrentino@crea.gov.it; 2 Department of Biology, Università degli Studi di Napoli Federico II, 80126 Napoli, NA, Italy; giordano@unina.it

## 1. Introduction

Since 1973, with the activation of the Environmental Action Program (EAP), the European Union has produced a substantial body of legislative packages aimed at improving the environmental quality; as a result, air, water, and soil pollution have significantly been reduced, as stated by the European Commission in the EAP 2020. Nonetheless, environmental pollution is still one of the most serious problems worldwide, particularly in densely populated and industrialized areas. Natural sources and anthropogenic activities release persistent pollutants, such as heavy metals, PAHs, dioxins, and microplastics, contaminating the air, water bodies, sediments, and soil. These contaminants pass from the abiotic components of ecosystems to the biotic ones, passing along the food chain and causing severe damage to the environment and living beings. Therefore, the extensive monitoring and removal/stabilization of pollutants from the environment represent important challenges to minimizing the damage. Plant organisms are currently used in both phytomonitoring and phytoremediation due to their intrinsic properties. Specifically, plants, which are sessile organisms, cannot escape environmental threats and have developed peculiar strategies to face environmental stresses [1]. Moreover, pollutants may induce plant sensitivity or tolerance, allowing them to be used in phytomonitoring (i.e., as bioindicators or bioaccumulators) or phytoremediation [2,3,4]. Phytominitoring allows the establishment of dense monitoring networks with costs relatively lower than classical monitoring devices. However, despite the widely consolidated approaches, such as the use of epiphytic lichens as bioindicators and moss and lichen bags, few data exist on the effect of urban greenery as a barrier to mitigate air pollution [5], or the potential of plants as biomonitors of microplastics [6,7], reflecting relevant gaps in the scientific knowledge. Regarding the remediation of polluted soil, the physical and chemical methods are generally expensive (10–50 times more expensive than the plant-based protocols) [8], and their application returns soils devoid of their original biological properties, sometimes producing new wastes. The use of the plants and associated microbiota in restoring polluted soils, water bodies, and sediments are effective and eco-friendly methods. They are receiving increasing attention from researchers and stakeholders, also raising the public awareness of environmental protection for people’s improved life quality.

## 2. An Overview of Published Articles

This Special Issue is a collection of eleven papers, nine research articles, and two reviews focused on phytomonitoring and phytoremediation. The review by Wani et al. (2023) (contribution 1) summarizes the phytoremediation techniques with a modern and updated approach, focusing on the use of soil microorganisms to stimulate the growth and uptake of pollutants by plants and the use of genetic engineering to exploit plant species with high-level biomass production, but a limited ability to uptake pollutants. The review by Elazab et al. (2023) (contribution 2) deals with plant–metal interactions, with special attention given to the in vitro studies. Controlled experimental conditions allow one to explore the mechanisms underlying metal uptake during plant developmental phases, as well as the regulatory substances and microorganisms that can improve metal absorption and translocation.

Several articles are focused on the in situ bioremediation of chronically contaminated sites through biostimulation. Hassan et al. (2023) (contribution 3) studied the ability of *Arabidopsis thaliana* to accumulate/stabilize arsenic and lead in polluted soil with different compost–biochar mixtures. The authors highlight that all the combinations stabilized Pb and mobilized As, but only the 20% compost–6% biochar mixture caused plant growth, suggesting that this approach is a promising protocol to reclaim As polluted lands. Udume et al. (2023) (contribution 4) present their results on the recovery of a petroleum-polluted soil by inorganic and organic amendments. In particular, the latter the application of compost from water hyacinth and spent mushroom gave the best results, showing that the 16 PAHs designated by the US EPA as priority pollutants were either completely or highly degraded, thereby indicating the potential of this amendment for the remediation of soils contaminated by recalcitrant organic pollutants. Garau et al. (2023) (contribution 5) studied the effect of compost, biochar, and their combination on soil polluted by Sb and Zn. The authors found that compost improves the nutrient mobility and soil enzymatic activities with a positive effect on *Lolium rigidum* growth, whereas biochar reduces the PTEs’ mobility probably due to its high absorption capacity.

Kudo et al. (2023) (contribution 6) studied the effect of temperature on the absorption, distribution, and removal kinetics of Cd and Zn in *Arabidopsis hallerii* grown in hydroponics. The authors highlight that although the two metals share similar transport pathways, different temperatures regulate their uptake and distribution, suggesting different loading capacities of the xylem for transport; this trait could help with Cd removal in matrices also containing Zn, such as Zn ores.

The phytoremediation potential of four native plant species growing in mine soil was investigated by Azizi et al. (2023) (contribution 7). The authors found that all the species were able to absorb/stabilize metal(loid)s using species-/element-specific behavior, but no species could translocate the REEs. Meister et al. (2023) (contribution 8) studied the potential of native Mirtaceae on the phytomanagement of pasture irrigated with treated municipal wastewater. The authors found that native Mirtaceae were able to accumulate more trace elements in their leaves, while N and P were taken up at higher rates in pasture grass. These results suggest that the irrigation with treated wastewater can take advantage of the presence of native species, with positive effect on nutrient uptake in the target plants (i.e., pasture grass).

A different aspect of phytoremediation is the effect of the pollutants on plant growth and physiology. This aspect was investigated by Sumalan et al. (2023) (contribution 9) and Liu et al. (2024) (contribution 10). In the first article, the effects of Cd, Cu, Pb, and Zn were tested on *Silphium perfoliatum* in hydroponics. The authors evidenced different uptakes and distributions depending on the metal, its concentration, and the plant organ considered (the root, stem, or leaves). Different accumulations also affected the enzymatic activities, pigment production, and proline content. Liu et al. (2024) (contribution 10) studied the accumulation of Cd in *Lonicera japonica* exposed to graphene oxide, which is a carbon-based nanomaterial that was recently used to improve the phytoremediation of metal polluted soil. In the test samples, Cd accumulation improved the plant’s photosynthetic performance (carbon sequestration and oxygen release) more than that of the control, with a positive effect on biomass production.

Finally, Postiglione et al. (2023) (contribution 11), present a novel article on next-generation biomonitoring. The authors investigated the microbiota associated with *Quercus ilex* leaves in urban and natural sites and analyzed the PM concentration and PAH content from the sampled leaves collected from the same sites. The authors found that microbiota biodiversity was related to the season, the anthropogenic impact, and the pollution level; these results suggest that the microbic communities associated with leaves may act as good bioindicators.

## 3. Conclusions

This collection of articles demonstrate the keen interest of the scientific community in the topic of environmental pollution and the setup of sustainable solutions used to monitor and recover ecosystems. Moreover this collection highlights the key role of the plants as model organisms, also as microecosystems hosting microbial communities. Integrated skills and approaches based on different expertise, disciplines, and methodologies are needed to delve deeper into all the aspects of phytomonitoring and phytoremediation. From this perspective, this Special Issue represents a valuable discussion among scientists to harmonize their results, set milestones, and provide new food for thought for the future.
